# Medical and Physical Intelligence

**Published:** 1801-10

**Authors:** 


### MEDICAL AND PHYSICAL INTELLIGENCE.

### *Dr.* HAHNEMANN'S *Preservative Remedy against the Scarlet Fever.*

In a former number of this Journal, we communicated to our readers a notice of a preservative remedy against the Scarlet Fever, discovered by Dr. HAHNEMANN, but at that time kept a profound secret. However adviseable it may be to distrust such arcana, by which the credulity of the public is frequently deluded, yet we meet sometimes with instances of their having proved very beneficial to the community, when the mystery in which they were involved is disclosed, and their use reduced to a just compass. The remedy, however, of which we are speaking, ought to have excited the suspicions of medical men, not so much on account of its inventor, who has acquired by his works a respectable character among the German physicians, as on account of the singular method of use, as it was ordered to be administered in doses smaller than could be reasonably presumed to have any effect on the human constitution, even if its powers were the most concentrated. *We shall not indulge ourselves in inquiries on the possibility and use of such a remedy,* but only give a short account of what Dr. HAHNEMANN has stated concerning his remedy. He has but lately satisfied the expectations of the public in a pamphlet, which is intitled, "*Heilung und Verhuetung des Scharlachfiebers,* i. e. Cure and Prevention of the Scarlet Fever, by Dr. SAMUEL HAHNEMANN, 1801." After having premised the description of an epidemical scarlet fever that fell under his care, in which he gives an accurate account of the general symptoms of this disease, and of their particular nature and succession in that epidemic, he relates his method of cure, which in most cases proved quite satisfactory. He is of opinion, that there are *two states* to be distinguished in the constitution at the period when the disease has fully taken place. The first, attended with a burning heat, sopor, uneasiness of the body, vomiting, diarrhœa, &c. is relieved in a short time by a very small dose of opium, either externally applied by putting a bit of paper moistened with a strong tincture of opium on the pit of the heart, or given internally. For the external application, he used a tincture consisting of one part of finely pulverised opium purum, dissolved in twenty parts of diluted spirit of wine, with which it was suffered to stand in the cold for a whole week, and the tincture frequently shaken. For internal use, one drop of this mixture was intimately mixed with 500 drops of very diluted spirit of wine; and of this mixture again, again, *one* drop in 500 drops of very diluted fpirit of wine. Of this very diluted tincture of opium, containing about $\frac{1}{5000000}$ gr. of opium in each drop, one drop was fufficient for a child of four years, and two drops for one of fix to ten years; which dofes were only to be repeated every four or eight hours, fometimes once in twenty-four hours; and in fingle cafes, once or twice during the whole difeafe. *Nothing will more certainly relieve the above symptoms than this mode of employing the opium, the effect of which may be always expected in one hour, and sometimes in only a quarter of an hour, after the application.*

*The second diseased state,* manifefting itfelf by the fever increafing towards the evening, by the entire want of appetite, naufea, an unfupportable peviſhnefs, is certainly relieved in lefs than half an hour by *ipecacuanha*; of which $\frac{1}{10}$ to $\frac{1}{2}$ of a grain is to be given at the commencement of this ftate of the body. A tincture of ipecacuanha may likewife be ufed for this purpofe, confifting of *one* part of ipecacuanha digefted for feveral days in 20 parts of diluted fpirit of wine, of which one drop is to be mixed with 100 drops of fpirit of wine, and the dofe is one drop for the youngeft, and ten drops for the oldeft child.

The principal part of the publication is devoted to the preventive remedy againft the Scarlet Fever, which is *the inspissated juice of the fresh leaves of the wild growing belladonna,* or *deadly nightſhade,* (*Atropa Belladonna, L.*) The author was firft led to this difcovery by the fimilarity of the fymptoms produced by the belladonna to thofe which arife during the fcarlet fever. He employed it accordingly; and as it exceeded his moft fanguine expectations, he thought it entitled to that praife with which he has made it fo publicly known. The remedy is prepared in the following way: Take a handful of the freſh leaves of belladonna, gathered before the flowering; after having bruifed them in a mortar, exprefs the juice through a linen bag, and pour it on china plates, where it is fuffered to evaporate till it be dry. Having pulverifed it, preferve it in fmall bottles that have been previoufly heated. One grain of this powder is diffolved in 100 drops of diftilled water; the turbid folution is put into a bottle containing one ounce, after having added 300 drops of fpirit of wine diluted with five parts of water, with which the mortar wherein the powder was mixed with the diftilled water has been waſhed. The bottle is marked, *Strong solution of Belladonna.* One drop of this is mixed with 300 drops of fpirit of wine, and the mixture ſhaken for fome minutes; it is marked, *Middle solution of Belladonna.* Of this fecond mixture, one drop is again combined with 200 drops of fpirit of wine, and marked, *Weak solution of Belladonna. This is the preservative,* which contains in every drop $\frac{1}{24000000}$ of a grain of belladonna juice. The dofe in which this remedy is adminiftered for the purpofe of preferving perfons from infection, is, For a child of one year, three drops; for one of three years, four drops; for one of four years, five drops; for one of five years, fix or feven drops; for one of fix years, feven or eight drops; for one of of feven years, nine or ten drops; for one of eight years, eleven to thirteen drops; for one of ten years, fourteen to fixteen drops; and then with every year two drops more: however, the dofe muft not exceed forty drops, for people of twenty or thirty years. It is repeated every feventy-two hours during the epidemic, and ought to be continued four or five weeks after. In very fevere epidemics, it is advifable to give the fecond dofe twenty-four hours after the firft; the third, thirty-fix hours after the fecond; the fourth, forty-eight hours after the third; and the following dofes every feventy-two hours. No peculiar regimen is to be obferved during the ufe of this remedy, except that the children muft take care not to catch cold, as the effect of the belladonna is much abated by it, and it is therefore proper to increafe the dofe of the remedy in this cafe.

The too frequent ufe of vegetable acids ought likewife to be avoided, becaufe the effects of the belladonna are too much increafed by it, according to the experience of Dr. HAHNEMANN. Any violent action of the belladonna that may accidentally take place, is fpecifically removed by the above method of employing the opium. In any violent depreffing affection of the mind, by which the tendency to infection increafes, it is thought proper to adminifter a few dofes of belladonna, befides the regular ufe, and children naturally peevifh fhould always take a double dofe of belladonna folution. The mixture ought to be moft intimately combined, and fhaken for a good while before it be taken; a circumftance that muft be carefully attended to.

Dr. HAHNEMANN fuppreffed a beginning fcarlet fever, with all its attending fymptoms, in the fpace of twenty-four to forty-eight hours by the ufe of belladonna, of which he ordered half the dofe above mentioned to be taken every three hours, till all fymptoms difappeared, and then he continued a whole dofe every feventy-two hours, in order to prevent a new infection. When the fcarlet fever is fully eftablifhed, Dr. H. diffuades the ufe of belladonna.

Any ill confequences that may fupervene after the fcarlet fever is over, are removed by the fame mode of giving the belladonna, except in fome fingle inftances, where it did not prove of avail, particularly in the remarkable difpofition of the fkin to exulcerations. In this cafe, however, he recommends the juice of chamomile, *matrica chamomilla,* infpiffated in the air without heat, as the fureft remedy. One grain of it is diffolved in 500 drops, and then intimately mixed with 500 drops of fpirit of wine, of which folution, one drop is dropped to 800 drops of diluted fpirit of wine, the dofe of which is one drop to a child of two years, &c.; and this muft be continued every day, till the cure is performed, for a few days. One drop of that folution contains about $\frac{1}{800000}$ of a grain of the infpiffated juice. This remedy is likewife of ufe in vehement coughs and a feverifh ftate, that are fometimes the confequences of a Scarlet Fever.

 PORTAL'S

PORTAL's *Observation of a Motion in the Spinal Marrow.*

It is known, that the motion of the brain keeps pace with refpiration, a phenomenon, that has been alfo obferved by Mr. PORTAL in the fpinal marrow. He faw a child born with a *spina bifida* at the upper part of the back-bone, in which the fwelling of the fpinal marrow could be clearly perceived at every expiration. On opening the body after the death of the child, he difcovered a canal in the middle of the fpinal marrow of the thicknefs of a pen, filled with a reddifh liquor, and communicating with the ventricles of the brain, that contained a fimilar liquor. On cautioufly opening the fpinal canal of new born dogs and cats at its upper part, the fucceffive fwelling and contraction of the fpinal marrow is manifeftly diftinguifhed to correfpond exactly with the motion of the brain. The motion of the fpinal marrow, however, feems only to take place at its upper part; and Mr. PORTAL could neither difcover it in the *spina bifida* of the lower part of the back-bone, nor in living animals, on opening the back-bone beneath the fourth vertebra colli, where the motion feems to abate and difappear. It is not improbable but this motion may continue in the natural ftate, the fpinal marrow being thinner than the cavity of the back-bone, and the motion can therefore freely go on. This, however, is not the cafe in the brain, which fills the cavity of the fkull fo exactly, that no fpace is left for allowing any fwelling, and therefore never takes place except when a hole exifts in the fkull, or when the bones are of a particular thin texture. The brain however would certainly fwell at every expiration by means of the blood veffels, and a compreffion of its fubftance be occafioned, if the venæ vertebrales did not convey the blood back, at which place therefore a fwelling is occafioned, which cannot take place in the brain itfelf for want of room. In this way a preffure on the brain is prevented, as otherwife we fhould more frequently be expofed to apoplexies.

---

*Dr.* SELIG, *on the Use of the Extractum Cardui Benedicti.*

This remedy is very much recommended in catarrhous complaints of children, and its virtues in all catarrhous difeafes feem to be almoft fpecific. It contains a confiderable quantity of nitre. Two fcruples of the extract are diffolved in feveral ounces of diftilled water, with the addition of 100 drops of effentia cortic. aurantiorum or effentia Scordii, of which mixture, 50 drops are to be given feveral times a day. The remedy is likewife very efficacious in rheumatic complaints, with fpiritus Mindereri, in the dofe of 60 drops every three hours. It is alfo of great ufe in pleurifies, after having undergone the proper evacuations; it diffolves any inflammatory obftruction, and promotes fecretions and the critical excretions by fweat, urine, and expectoration. He farther praifes it in feveral kinds of fore throat, exanthematous difeafes, fcrophula, glanduluar glandular indurations, obftructions of the vifcera, icterus, &c. in all which cafes it ought to be combined with proper remedies.

---

We find the ufe of *Mesembryanthemum cryftallinum, L.* an annual plant of the fucculent tribe, very much recommended by Prof. Wendt, of Erlang, particularly in difeafes of the urinary fyftem. Its medical qualities were firft made known by Dr. Lieb, of Mitau, in a particular pamphlet, entitled *The Ice-plant,* (Eispflanze, which is the name it bears in Germany) *recommended almoft as a fpecific remedy, by D. J. W. T. Lieb, practitioner at Mitau, Hof.* 1785, (German) in which the juice of it is highly praifed in difeafes of the urinary fyftem, hooping-cough, accumulation of pituita, and bilious complaints. It feems however to have been much difregarded by practitioners; for, except in Murray's Apparatus Medicaminum, Vol. VI. p. 130; by Fuchs, in Crell's Chemical Annals, 1787, Vol. I. p. 503; Hoffmann, in Almanac for Chemifts and Apothecaries, 1788, p. 81, who both give an analyfis of the plant; Loefeke, Materia Medica, Ed. VII. Gmelin, we find it no where mentioned. Dr. Lieb fays, that the juice taftes like terra foliata tartari, and he thinks that it contains nitre, though he had not a fufficient quantity for analyfing it. He gave the juice in a dofe of a table fpoon full once a day to adults, and half as much to children; and he obferved, that the urine depofited a more or lefs confiderable fediment; with refpect to its diuretic qualities he prefers it even to fquill, and thinks it might be employed with fuccefs in cacochymies, dropfies, confumptions, fevers, &c. He relates four cafes in which this remedy proved feviceable, three of which were bilious complaints, and one hooping cough; but as he ufed other remedies at the fame time, the obfervations are not fo much to be depended upon. According to the experiments of Mr. Fuchs and Hoffman, the plant contains feveral neutral falts; and the latter obtained from fix pounds of the leaves, the following conftituents:

| | |
|---|---|
| Oil - - - - - - - - - - - - - | 48 grains |
| Sal ammoniac - - - - - - - | 7 |
| Sal digeftivum - - - - - - - | 44 |
| Potafh - - - - - - - - - - - | 153 |
| Nitre - - - - - - - - - - - - | 87 |
| Vitriolated tartar - - - - - - | 1 |
| Selenite - - - - - - - - - - | 6 |
| Alum - - - - - - - - - - - | 14 |
| Salt, of a fingular figure like grains, and tafting as nitre, - - - - | 8 |

The tafte of the plant is cooling, and when eaten as a fallad it acts purgatively, and caufes likewife a fediment in the urine. Dr. Wendt relates feveral cafes in which he has tried the juice of the above plant with fuccefs. The firft was a patient, who, befides complaints in the bowels, was afflicted with a painful difficulty on making water, for which feveral remedies were given without relief; having at laft obtained a fufficient quantity

 of of the plant, it was accordingly employed, and with fuch unexpected fuccefs, that the patient got free, after a fhort time, of that difagreeable affection, by its effecting the fecretion of a great quantity of pituitous, ill fmelling matter, that iffued from the bladder. Another patient, who from a conftriction in the urethra, fuppofed to be occafioned by a fwelling of the proftate, was afflicted with an obftinate ifchury, which refifting a great many other remedies, was removed by the internal ufe of the juice of *Mesembryanthemum cryftallinum, L.* It was likewife found to be of effect in the enurefis fpaftica, in which the urine goes infenfibly off by drops, and of which the author alfo relates two cafes. The juice of this plant is an excellent refrigerant, in mitigating the febrile heat, a fymptom which fo exceedingly molefts the patients in hectic and confumptive fevers. The Profeffor employed it likewife with fuccefs in feveral cafes of hooping cough.

---

*Professor* ROOSE, *of the Power of the Will on Respiration.*

Refpiration is generally confidered as a voluntary motion. The fuffocating fenfation which we feel, when we endeavour to continue in a ftate of infpiration or expiration, is faid to determine our will to change thefe ftates alternately. But in order to make the continuance of refpiration during fleep agree with this explanation, as that phenomenon feems to contradict the general opinion, refpiration was called a *motio mixta,* without ever regarding by what caufe that function acted, when the will ceafed to influence it. Profeffor ROOSE of Brunfwick, propofes in a letter to Profeffor REIL of Halle, a conjecture refpecting this fubject, which we think worthy of the attention and inquiries of Phyfiologifts.—After having fhortly proved the impoffibility of merely explaining the continuance of refpiration during fleep, and in epileptic and apoplectic fits, from the influence of the will on the organs of refpiration, it appears to him, that it muft be effected by another ftimulus in the body; and he propofes, therefore, the queftion, By what change in the body is the neceffity of the alternate infpiration and expiration produced? With refpect to this queftion, which deferves to be farther difcuffed by phyfiologifts, he is inclined to think, that *the motion of the brain* might throw much light on it.

This phenomenon, which has not yet been fo much attended to as it deferves, does by no means depend on the pulfation of the arteries, as far as we know, from the obfervations of BLUMENBACH, who could exactly diftinguifh that motion from the pulfation of the arteries in a young man of 18 years of age, who had loft part of the fkull. Mr. ROOSE is of opinion, that there might perhaps exift a caufal union between the refpiration and the motion of the brain. At each expiration the blood accumulates itfelf in the brain, and caufes an intumefcence and a ftate of increafed excitation, which, when continuing too long, produces a fuffocating fenfation, but not otherwife. The brain, which is in this this way irritated, begins to re-act on the organs of respiration, and thus occasions inspiration, which consequently does not arise from the will, but immediately originates in the change of the action of the brain. On the ensuing inspiration, the intumescence of the brain decreases, and the state of irritation ceases, the muscles of the organs of respiration are relaxed, and thus follows again expiration.

This ingenious idea ought to be farther pursued and ascertained by repeated observations, because it appeared from the experiments of ABILGAARD, that the oxydation of blood seems not to be the only effect intended by respiration, but that there is still something left unexplored in that function. It is therefore more than probable that the alternate and regular motion of the brain may be intimately connected with it, and not merely effected by the influx and reflux of the blood, as is generally imagined.

---

### VAUQUELIN'S *Method of analyzing Minerals.*

M. VAUQUELIN, to whose skill we are indebted for the analysis of many minerals, describes his method in the following way: The mineral, after having been pulverised, is mixed with three times as much potash, and melted in a crucible, and afterwards put into water, in which the mixture dissolves. For discovering whether any of the eight simple earths be contained in the mass, we ought to proceed as follows:

1. *The Siliceous earth* is dissolved in caustic alkalis, particularly in heat, and precipitated by acids; but again dissolved by a superabundance of them, a solution of this earth, in acids, appears like a gelatine. After evaporation, and when dry, it becomes insoluble in these menstrua, by which means we are enabled to separate it from other earths. In this state it is white, granulous, and dry to the touch, and perfectly insoluble.

2. *Argillaceous, or aluminous earth,* is likewise soluble in fixed alkalis and acids, from which, however, it cannot be separated by evaporation; it retains the water, to which it has a great affinity, and its particles concrete in heat, in which state the earth is white, half transparent, and sticks to the tongue. Its combination with sulphuric acid produces, by the addition of a few drops of sulphat of potash, octaedral crystals of alum.

3. *The Zirkon or Sargon earth,* is not affected by caustic alkalis, but acids dissolve it when it is pulverised; it must not, however, be previously calcined. It forms with sulphuric acid an indissoluble salt, but very slightly adheres to the other acids, from which it is separated by a moderate heat. When very finely pulverised it dissolves in carbonised alkalis, which are perfectly saturated with carbonic acid. In its pure state it is of a yellow colour, half transparent, and very brittle, much like gum arabic; but, after being calcined, it is white, opaque, and rough to the touch, and very difficultly soluble in water.

4. *The Glucine or Sweet earth,* dissolves like the Argillaceous earth in acids and alkalis, but more readily in carbonat of ammonia; it yields yields no alum with fulphuric acid and potafh. The falts which it forms with acids are fweetifh. In a dry ftate it is very white, light, and foft, and without any tafte. Its particles concrete in heat like argil.

5. *Magnefia* unites itfelf with all acids, forming with them very foluble and bitter falts. It is not perfectly precipitated from its folutions either by the carbonate of potafh or by ammonia. It is by no means foluble in cauftic alkalis, but has much affinity to alum. In its pure ftate it has a white colour and no tafte, and is very light and indiffoluble in water.

6. *The Calcareous earth, or Lime,* combines itfelf with acids, with which it yields partly foluble and partly infoluble falts. It does not diffolve in alkalis, and its folution in water is made turbid by carbonic acid, but not by fulphuric acid. It precipitates all preceding earths from their folutions, and is itfelf precipitated from its folutions by ammonia. In its ftate of purity it is of a fharp and burning tafte, and ferments with water, but the folution never cryftallifes.

7. *The Strontian earth* eafily enters into a combination with acids, and forms with fulphuric acid a very foluble falt. It diffolves plentifully in warm water, which folution fhoots into beautiful cryftals fimilar to fal ammoniac. Selenites produce a precipitation in its folution, which, when combined with muriatic acid and dif-folved in alkohol, burns with a blue flame. This earth has a fharp tafte, and ferments with water.

8. *Barytes, or heavy earth,* poffeffes many properties in common with the Strontian earth, from which it is not to be diftinguifhed but by being more foluble in cold water, and by its combination with muriatic acid being very little foluble in alkohol. It cryftal-lifes when the folution becomes cool. Its tafte is fharp, and it ferments with water; it forms with fulphuric acid an infoluble falt, and decompofes the alkalis that are combined with fulphuric and carbonic acid like the ftrontian earth; which procefs is attended with fuch phenomena as are not perceived by thofe who have no great experience in chemical proceedings.

---

Mr. Boeckmann has made feveral experiments refpecting the particular influence of light on phofphorus diffolved in different gafes. It has always been a matter of enquiry and difcuffion, whether there is any real difference between heat and light, fome Naturalifts having attempted to prove the identity of thofe two matters; while others maintained, that there exifted a great diverfity between them. From the experiments of Mr. Boeckmann, however, it feems to refult, that dark heat, and heat combined with light, produce phenomena effentially differing from each other; and that, accordingly, we ought not to adopt an identity in the nature of heat and light. As it would lead us too far to relate the fingle experiments, we fhall only enumerate the facts that have a-rifen from them, and which are the following.

1. The folution of phofphorus in hydrogen gas proceeds not only at a heat combined with light, but alfo at a dark heat.

2. It

2. It proceeds with more fpeed when the temperature is increafed, whereby, at the fame time, a greater quantity of phofphorus diffolves than at a lefs degree of heat.

3. When the folar light is kept off by opake bodies, either folid or fluid, from the glafs tubes in which the phofphorus is expofed to folution, no phofphoric particles are found to adhere to the fides of the tubes, even when the degree of heat is fo confiderable as to melt the phofphorus; whereas,

4. When direct or reflected folar light falls on the glafs tubes, the phofphorus is, as it were, fublimed at the fides of the tubes.

5. The phofphoric particles lay themfelves on the inner fides of the tubes more or lefs quickly, according to the degree in which light acts upon them.

6. Thofe fublimations of phofphorus are even produced when the light is faint.

7. When the glafs tubes are covered on the outfide with opake ftripes, alternately with places uncovered, the phofphorus only adheres at thefe fpots.

8. The difference of temperature has no influence on the phofphorus adhering to the places where light acts.

9. The experiments were made in veffels very clofely fhut, fo that the atmofphere had not the leaft fhare in producing the cryftallizations of phofphorus that adhered to the inner fides of the tubes.

On the whole, in thefe experiments we meet with two phenomena, viz. the folution of phofphorus in gafes, and the adhefion of it to the fides of the veffel in which the folution was undertaken, when they are expofed to the light of the fun. The folution is undoubtedly promoted by heat, though the adhefion never takes place without the action of light. But how this is caufed, is not fo eafily to be refolved.

---

According to the obfervations of Prof. Lampadius, water is contained in foffils in three different ways. 1. Water that only adheres to them; 2. Water of cryftallization, which goes off at the burning of the foffils; 3. Water that is fo intimately combined with them, that it can only be feparated by the chemical affinity of feveral fubftances during the procefs of melting. Burn, for inftance, quartz, porcelaine earth, and fpathum calcareum, every one fingly, till no more water goes over; mix them together, and melt them, during which procefs, a confiderable quantity of water will be ftill feparated.

---

The diftilled water of *Prunus Laurocerasus, L.* is a remedy that belongs to the poifonous fubftances, and it ought, therefore, to be employed with great care and prudence. The obfervations remarked by Cameron, Thilenius, &c. on the virtues of that remedy, have induced Prof. Wurzer to make new trials with it on animals; the refults of which fhow, that it diminifhes the force of the the heart, and the irritability of the mufcular fibres; and that it brings the abforbent fyftem into a greater action. The author prefcribed it in a cafe of hypochondriafis, in the dofe of fifty drops, to be taken three times a day; by which remedy the patient was completely cured. It is, certainly, very powerful and active, and may prove of great effect in the hands of an able and prudent practitioner.

---

Mr. SCHUMACHER has found the Fabæ pichurim very efficacious in the fluor albus, particularly when this difeafe was inveterate, or owing to debility. The dofe in which he gave it, amounted to three fcruples given three times a day. After having taken from twelve to twenty-four dofes, the patients found themfelves greatly relieved.

---

The fame gentleman employed the Cortex caribæus in feveral kinds of intermittent fevers, but without effect. It feemed to be of fome fervice in rheumatic complaints, by promoting diaphorefis. He never remarked any emetic quality on ufing it. It was given from half a drachm to one drachm in powder, every two hours, or in a decoction of unc ij. to one pint and a half of water, of which every two hours, half a cup full was to be taken.

---

Dr. VAN STICHELL has made feveral obfervations on the Cachexy, produced by the abufe of fpirituous liquors, and on its proper method of treatment. The habit of drinking fpirits, fo familiar to the northern nations, gives rife to a difeafe known by the name of cachexy, the immediate caufe of which, feems to originate in an obftruction of the *ductus excretorii* of the falival and mucus glands, which occafions a congeftion of mucus matter in the glands, and a general morbid fwelling, attended with total lofs of appetite, perpetual thirft, and a general apathy, which at laft terminates in death. For curing this difeafe, the patient is obliged to abftain from fpirits, his ftrength muft be kept up by nourifhing broths; and for refolving the obftructions, he prefcribes the waters of Seltz, either pure, or with a little red wine, which effect he expects from them, as they contain carbonat of lime and magnefia.

---

Cit. BERTHOLLET has made feveral obfervations relating to Eudiometry, in a memoir prefented to the National Inftitute of France. The celebrated Mr. Humboldt having examined and compared the different fubftances employed for knowing the quantity of oxygen contained in a given quantity of atmofpherical air, gave the preference to the nitrous gas, which has been propofed by Fontana, as abforbing the oxygen better than other fubftances employed for the fame purpofe. It contains, however, fometimes times a greater or lefs quantity of azote, which renders its effect not fo fure; a circumftance which may be remedied, after the repeated experiments of Mr. Humboldt, by wafhing the gas intended for ufe with a folution of fulphat of iron, which abforbs all the nitrous gas, but not the azote that is mixed with it. It is this action of the fulphat of iron that Cit. Berthollet has examined; and he has found, that in this cafe, the nitrous gas is not only abforbed, but decompofed, and by being deprived of a part of its azote, it is changed into nitrous acid. He thinks, on the whole, that the nitrous gas may differ in the proportions of its two principles, the oxygen and the azote, but that it never contains the azote in fimple mixture.

---

Cit. PORTAL has communicated fome ideas on epilepfy, its caufe, and feat in the brain, that is either primarily or fecondary affected in this difeafe of the nervous fyftem. This practitioner adopts two diftinct fpecies of epilepfy; according to thefe two kinds of lefion of the brain, in the fecond cafe of which, when the fpafmodic ftate and the pain proceed from one part, and particularly from any external part towards the brain, the methods of cure are generally employed with fuccefs. Cit. Portal has ufed, in two inftances of this kind, topical anti-fpafmodics, with much effect, applied and rubbed in at the place whence the fpafm and the pain feemed to proceed, one hour before the paroxyfm came on. Thefe two facts are, indeed, worthy the attention of practitioners, particularly as we have analogous obfervations of convulfive difeafes of the fame nature being cured by cauteries luckily applied, by ligatures, accidental burnings, &c.

---

*On the Faba Pichurim, and its constituent Particles, by* J. E. F. KOBES, *Apothecary at Berlin.*

The *Faba Pichurim* or *Pecurum,* which has formerly been much ufed againft dyfenteries and diarrhœas, but is now obfolete, is the fruit of a tree that grows wild in Paraguay, in South America. Mr. BERGIUS refers it to the genus Laurus, and gives to it the name of *Laurus pechurim*; which Mr. SCHREBER however oppofes, as the fruit is quite different from that of a laurus. There are the *Faba Pichurim, major and minor*; the firft of which is of an oval form, about one inch to an inch and a half long, on its inner fide concave, on the outer fide convex. Its furface is of a brownifh colour, but it contains infide a reddifh, friable fubftance, of a fragrant fmell like nutmeg, and they have a bitter and fomewhat aftringent tafte. The little Faba Pichurim is fomewhat like the major, but not of fo elliptic a form, and its interior fubftance is of a dark brown colour. From their fhining and fmooth furface, I concluded that they contained a grofs oil. Twelve ounces of the great Faba Pichurim were accordingly reduced to a coarfe powder and preffed. but I endeavoured in vain to obtain an oil from them; I undertook however the fame operation with the Faba Pichurim minor, and obtained by that means from twelve ounces of the powder of the Faba

 Pichurim

Pichurim minor, about five drachms and a half of a grofs oil, having the confiftency of Cakoa butter. The weight of the bag in which the preffure had been undertaken, was found to be augmented by four drachms and a half, after being cleared from the powder, fo that twelve ounces of the fmall Pichurim bean yielded about ten drachms of this grofs oil, which has quite the fmell of the bean, and eafily concretes in the cold air, and has a greyifh furface. For obtaining the effential oil of the great Pichurim bean, I put ten ounces of them, previoufly powdered, into a retort, and having added a fufficient quantity of water, I diftilled them at firft with a gentle heat, which was by degrees increafed. The procefs being over, a brownifh oil was found to fwim on the furface of the water, which being again expofed to diftillation, four fcruples of a light yellow oil were obtained. I undertook the fame operation with the fmall Pichurim bean, but the quantity of effential oil was found to be much inferior in this bean than in the other; whence it appears, that the Faba Pichurim major contains a greater quantity of effential oil than the Pichurim minor, that is richer in grofs oil. Though it would not be proper to employ this remedy in form of an extract, as its effects feem chiefly to depend on the effential oil, yet I made extracts of both fpecies of beans, in order to know the proportion of extractive fubftance that is contained in them. Of eight ounces of the great bean, I obtained four ounces and two drachms of a bitter, fomewhat aftringent extract; and of the fame quantity of the Pichurim minor, I feparated two ounces and three drachms of a very mucilaginous extract. The proportion of aftringent matter is not very confiderable in both fpecies of Pichurim beans. For therapeutical ufe it ought to be employed in fubftance, or, at leaft, in a fpirituous effence. The Faba Pichurim was formerly given in powder to ten grains, and chiefly recommended in dyfenteries and diarrhœas by Heuermann.

---

Prof. Hufeland has made feveral experiments by applying a folution of phofphorus in oil, or naptha vitrioli, externally, in frictions, which were of very great fervice in obftinate rheumatic and arthritic pains, and in venereal *dolores artium* and *offium*; paralytic complaints, and a venereal *tumor* were much diminifhed by it.

---

The fame gentleman mentions two inftances where the daily ufe of fweet almonds, together with a few bitter ones, had cured the tape-worm, which had refifted the moft efficacious remedies. If this was proved by farther experiments, it would be very beneficial, particularly for irritable perfons.

It is well known that the troublefome afcarides refift many powerful remedies. In one cafe, however of a man who was very much plagued with thefe animals, a tea cup of lime-water was applied by a clyfter, the fuccefs of which fimple remedy exceeded all expectation, and anfwered the purpofe better than any remedy that had been previoufly ufed.

Cit. Bichat

---

Cit. Bichat thinks, that the caufe of the arteries being found empty *after death*, ought to be afcribed to the reforption continuing fome time after; becaufe we frequently find in them fibrous matter, but no ferum. If this phenomenon arifes, as it is generally believed, from the power of the arteries in propelling the blood ftill continuing after death, and from want of refiftance in the veins, it cannot be well explained why the fibrous matter fhould not quite as well enter the veins as the ferum. Hence it feems probable, that a part of the blood remains in the arteries, where it is decompofed, and its ferous part abforbed by the lymphatics that terminate in the interior membrane of the arteries. A fimilar procefs moft likely takes place in the heart, in which we alfo find much fibrous matter without ferum.

### *Cow-Pox in the Sixth Century.*

In a paffage where Marius, firft Bifhop of Laufanne, author of Annals of His Own Time,* mentions the pox, *Variola,* (there was only one fort at that time) he obferves, that it principally attacked horned cattle. It even appears, that it only attacked mankind the year afterwards, namely, in 571 :† From this it is evident, that cows were fufceptible of it. It will turn out very fingular, if the fame animal which firft had this difeafe, fhould furnifh man with the beft preventive of this dreadful malady. It is furprifing, that this difeafe has fcarce ever been obferved amongft cows fince this very ancient æra.

Dr. S. H. Jackson propofes to publifh in the courfe of the enfuing winter, the Firft Part of a work, entitled, "*Elements of Clinical Practice*," confifting of Medical Hiftories and Obfervations, felected from the records of his attendance on upwards of 24,000 patients of the Weftminfter General Difpenfary, from the commencement of the year 1780 to the prefent time.—The principal defign of this work is to communicate a body of ufeful practical information, concerning the moft important fubjects in the general practice

* Marius is the oldeft French hiftorian; he was firft Bifhop of Aventicum, afterwards of Laufanne: He died in 596.

† "Anno 570. Hoc anno morbus validus cum profluvio ventris et *variola* Italiam Galliamque valde afflixit. Et animalia bubula per ea loca maxime interierunt.—Anno 571. Hoc anno infanda infirmitas et glandula, cujus nomen eft puftula, in fupra fcriptis regionibus innumerabilem populum devaftavit.

Muller, in his Hiftory of Switzerland, Book I. page 132, &c. quotes a paffage from *Paul Warnfrid*, agreeing with the above, where he fpeaks de glandulis in modum nucis quas fequebatur febrium æftus; and another from Anastasius, who fpeaks de percuffione fcabierum, ut nemo poffet mortuum fuum internofcere; which, according to *Müller*, agrees with the Small-pox, and which, at firft, as well as the venereal difeafe, was more terrible and fatal in its confequences than others, becaufe they were ignorant of the method of cure at this epocha.

practice of medicine, pointed out in the histories of particular cases, with occasional remarks and illustrations.

Dr. BRADLEY, (removed from *Delahay Street* to *Parliament Street*) will commence his Autumnal Course of Lectures on the Theory and Practice of Medicine, on Tuesday, October 6, at the Lecture Room, No. 102, Leadenhall Street, at six in the afternoon. On one or two evenings in the week, during the season, a Course of Lectures on the Principles and Application of Chemistry will be delivered by a Practical Chemist. The Experiments will be made in the presence of the Pupils.

Dr. BATTY's Lectures on Midwifery will comence on Monday, Oct. 5, at eleven o'clock, in Great Marlborough Street.

Mr. CHEVALIER purposes giving his Lectures on Surgery this season, at the Dispensary in Gerrard Street, Soho, instead of his own house, as was stated in our last Number.

