# Errata

**Published:** 1801-11

**Authors:** 


### ERRATA.

Page 374. l. 16, *for* by ammonia, *read* by carbonat of ammonia.

l. 20, *for* foluble, *read* infoluble.

